# P-2117. ETEST® Aztreonam-Avibactam (AZA) for Antimicrobial Susceptibility Testing of *Enterobacterales*: Performance Results From a Muticenter Study

**DOI:** 10.1093/ofid/ofae631.2273

**Published:** 2025-01-29

**Authors:** Sarah Prudhomme, Louise Bossy, Edith CSIKI-FEJER, Dwight Hardy, Mandy Wootton, Melanie L Yarbrough, Edwige Pillon, Christine Franceschi

**Affiliations:** bioMérieux, Marcy L'Etoile, Rhone-Alpes, France; bioMérieux, Marcy L'Etoile, Rhone-Alpes, France; bioMérieux, Marcy L'Etoile, Rhone-Alpes, France; University of Rochester Medical Center, Rochester, New York; Public Health Wales, Cardiff, Wales, United Kingdom; Washington University School of Medicine in St. Louis, St. Louis, Missouri; BIOMERIEUX, La Balme Les Grottes, Rhone-Alpes, France; BIOMERIEUX SA, La Balme les Grottes, Rhone-Alpes, France

## Abstract

**Background:**

AZA is a combination of Aztreonam, a monobactam not hydrolyzed by metallo-β-lactamases, and Avibactam, a novel non-β-lactam β-lactamase inhibitor with an extended spectrum that protects Aztreonam for hydrolysis by Ambler class A, class C, and some class D serine β-lactamases. Aztreonam-Avibactam is developed by Pfizer for the treatment of infections caused by Gram-negative bacteria, including metallo-β lactamase producers.

This study evaluated the performance of ETEST^®^ AZA (bioMérieux Marcy-L’Etoile, France), a new gradient diffusion strip, for determining antimicrobial susceptibility of *Enterobacterales* compared to the CLSI/ISO-20776-2 broth microdilution reference method (BMD).

**Figure:**
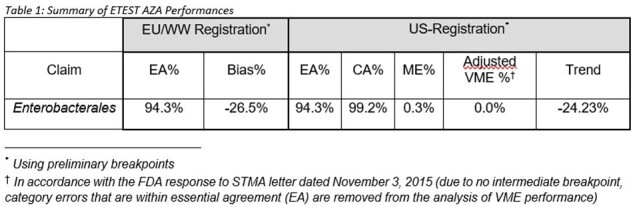

**Methods:**

A population of 650 (clinical and challenge) *Enterobacterales* isolates (including 150 *Escherichia coli;* 118 *Klebsiella pneumoniae*; 48 *Klebsiella oxytoca;* 67 *Enterobacter cloacae* complex; 50 *Citrobacter freundii* complex; 30 *Citrobacter koseri;* 32 *Klebsiella aerogenes;* 30 *Morganella morganii;* 32 *Proteus mirabilis;* 30 *Providencia stuartii ;* 30 *Proteus vulgaris ;* 33 *Serratia marcescens)* were tested at 4 clinical trial sites, comparing ETEST^®^ AZA and the BMD reference method.

Results were analyzed using preliminary EUCAST breakpoints (*Enterobacterales*: ≤ 8mg/L (S), > 8 mg/L (R)) and preliminary FDA breakpoints (*Enterobacterales*: ≤ 8mg/L (S), ≥ 16 mg/L (R)) provided by Pfizer. Results are presented as essential agreement (EA) and Bias for Worldwide registration and as essential (EA) and categorical (CA) agreements, Trend, very major (VME) and major (ME) error rates for US registration.

**Results:**

Results are summarized in Table 1. ETEST^®^ AZA performance for *Enterobacterales* met ISO acceptance criteria for EA (≥ 90%) and Bias ≤ ±30%, and FDA criteria for EA, CA (≥ 90%), ME (≤ 3%), VME (≤ 2%) and Trend ≤ ±30%.

**Conclusion:**

Results of this multicenter trial support the accuracy of ETEST^®^ AZA for determining MICs of *Enterobacterales*. As such, the ETEST^®^ AZA test is considered to be equivalent to BMD.

**Disclosures:**

Melanie L. Yarbrough, PhD, bioMerieux: Grant/Research Support|bioMerieux: Honoraria|Pattern Bioscience: Grant/Research Support|Shionogi: Advisor/Consultant

